# Cues derived from facial appearance in security-related contexts: a biological and socio-cognitive framework

**DOI:** 10.3389/fnhum.2013.00204

**Published:** 2013-05-16

**Authors:** Christopher D. Watkins

**Affiliations:** Division of Psychology, School of Social and Health Sciences, University of AbertayDundee, UK

Failures in the security process can have profound costs for both the individual and organizations (e.g., fraud costs the British economy approximately £72 billion; NFA, 2012). A biological and socio-cognitive framework may enhance our understanding of the security process, as the two perspectives collectively acknowledge that (i) competition for resources is/was an important factor in human social behavior and evolution (e.g., Bowles, 2009) and (ii) individuals differ in the ways in which they interpret information given their own traits and circumstances. Both levels of explanation (Mayr, 1963; Tinbergen, 1963) could generate novel hypotheses. For example, proximate-level explanations may clarify *how* resources are defended and extorted, and the cognitive processes underlying the “chess game” between gatekeepers and “gate crashers.” Ultimate-level explanations may clarify *why* some individuals are more likely than others to succeed at securing or gaining access to resources and whether certain security-related outcomes can be reliably predicted given specific contexts or ecological conditions.

## Decision-making under uncertainty

Humans make many decisions (consciously or otherwise) based on uncertain outcomes. Error management theory proposes that cognition has evolved so that when faced with two alternate strategies, we pick the strategy that would result in the least-costly errors (Haselton and Buss, 2000; Haselton and Nettle, 2006). This “cost-benefit” approach to decision-making is of value in describing the nature of human conflict. For example, sex differences in aggression are said to reflect the greater net “pay-off” to the reproductive fitness of males who engage in potentially risky competition for resources (see Archer, 2009 for discussion). Local differences in income inequality are also an important predictor of violent male–male competition (Daly et al., 2001), and may “pay-off” if harsh environments promote risky behavior in light of future economic uncertainty (e.g., Wilson and Daly, 2006). Research on environmental differences in behavior could provide an evidence-base for effective investment in crime-prevention (e.g., examining the local distribution of CCTV cameras), given that current strategies may be suboptimal (see, e.g., Webster, 2009).

Psychological mechanisms also play an important role in conflict. Overconfidence, the illusion of thinking you are better than you are, is an important cause of warfare (Johnson et al., 2006) and is more likely to evolve in contexts where the perceived benefits of competition outweigh their perceived costs (Johnson and Fowler, 2011). Indeed, this is neatly illustrated by George Bush's “mission accomplished” speech aboard the USS Abraham Lincoln in May 2003. Thus, given knowledge of context, ultimate levels of explanation can aid our understanding of the maladaptive practice of warfare.

Error management theories suggest that we will tolerate “false alarms” in circumstances where they are much less costly than having no alarm in place when really needed. Given the costs of security failure, to what extent will a gatekeeper tolerate false alarms (e.g., risk making a false conviction) given their own personality or immediate environment? These issues are clearly very current, as commentators debate the “trade off” between security and civil liberties. Indeed, the extent to which human error accounts for the false conviction of suspects (e.g., the innocence project; see Jenkins and Burton, 2011 for related discussion) is a neat illustration that demonstrating scientific evidence for a given behavior (e.g., cognitive errors/biases) is not the same as morally-endorsing that behavior (see, Greene, 2003).

Contextual cues may alter the nature of the trade-off between the perceived costs and benefits of identifying, controlling and monitoring perceived threats to the security of one's resources. Across species, evidence for the “winner effect” suggests that future decisions to engage in competition are modulated by recent experience such that winners more likely to escalate a future confrontation (even with a rival of higher rank than themselves) and losers are more likely to withdraw from future confrontation [reviewed in Hsu et al. (2006)]. Recent work suggests that confrontation outcomes modulate competition-related perceptions in men in a similar way as it appears to do in other species. Men who are primed to imagine having lost a confrontation find facial cues of dominance in other men to be more salient than men who are primed to imagine having won a confrontation (Watkins and Jones, 2012). These effects may be adaptive if they function as a compensatory response to the increased vulnerability of loss of resources in light of recent experience, and are consistent with other work which demonstrates how a lack of power can predict general inhibition in behavior and greater orientation toward threat [reviewed in Keltner et al. (2003)]. Contextual factors relevant to competition may predict systematic variation in judgments toward other cues of threat, such as facial expression or movement. Differential treatment toward others based on their appearance suggests an underlying biological basis to social interactions that might be important for effective competition.

## A biological basis to social judgments

Information provided by the face plays an important role in social interaction (Bruce and Young, 1986), and the categorization (e.g., Hugenberg and Bodenhausen, 2003; Mason et al., 2006) and identification (e.g., Hancock et al., 2000) of other people. We appear to be very quick to make our mind up about the character of an individual based on his or her facial appearance; trait judgments of faces made after just 100 ms of exposure are highly correlated with judgments made at longer exposure intervals (Willis and Todorov, 2006). A principal components analysis of trait judgments made toward faces revealed that differences in human face shape can be modeled on two primary dimensions, reflecting the extent to which an individual appears *intent* on causing harm to others (their perceived trustworthiness) and the extent to which an individual appears *capable* of causing harm to others [their perceived dominance; (Oosterhof and Todorov, 2008)]. Rapid judgments of traits that are important for personal safety are functionally adaptive if the costs of erring on the side of optimism are much greater than the costs of erring on the side of caution—the speed of social judgments at zero acquaintance may be more important than their accuracy [reviewed in Todorov et al. (2008)]. For example, given the potential costs of competition (Manson and Wrangham, 1991; Bowles, 2009), a rapid attribution of “threat” that turns out to be inaccurate is much less costly than an attribution of “no threat” that turns out to be inaccurate.

Perceptions of dominance and trust appear to have an underlying biological basis and are of obvious relevance to security scientists. Although the relationship between hormones and facial appearance is complex (see Pound et al., 2009), sex differences in the human face are thought to depend on exposure to gonadal steroids (see Puts et al., 2012 for discussion). Masculine physical characteristics in men are positively correlated with their perceived dominance (e.g., Perrett et al., 1998; Puts et al., 2006; Jones et al., 2010) and untrustworthiness (e.g. Perrett et al., 1998; Boothroyd et al., 2007). These attributions toward physically dominant individuals may have a “kernel of truth.” For example, physically dominant men are more likely to endorse the use of physical force to resolve conflict (Sell et al., 2009), are more aggressive in certain contexts (Carré and McCormick, 2008; Carré et al., 2009) and are less likely to share resources fairly with others (Stirrat and Perrett, 2010; Price et al., 2011) than their less dominant peers. From a biological perspective, physically dominant individuals should express less concern for the welfare of others than their less dominant peers, given that dominant individuals are better-placed to exploit or forcefully acquire resources with impunity (Sell et al., 2009; Puts, 2010; Stirrat and Perrett, 2010). Indeed, the costs of conflict are rarely symmetric between two parties (Maynard Smith and Price, 1973), and recent work suggests that facial cues of dominance in potential rivals are more salient to those who are less well-equipped to “offset” these costs (Watkins et al., 2010a,b). Systematic variation in dominance perceptions may be adaptive if it functions to minimize the costs of conflict in light of the perceiver's own dominance (Watkins et al., 2010a,b; Watkins and Jones, 2012). Exploring the extent to which the gatekeeper's own dominance predicts security-related outcomes may be a practical application for this line of reasoning.

Other aspects of facial appearance may predict trusting behavior in the exchange of resources. While attractive individuals are more likely to be trusted in economic exchanges (Solnick and Schweitzer, 1999; Hancock and DeBruine, 2003; Wilson and Eckel, 2006; Andreoni and Petrie, 2008), particularly attractive individuals are more likely than their less attractive peers to “shift” toward more trusting behavior when they believe that others' have the opportunity to take their appearance into account (Smith et al., 2009). Given that attractiveness is associated with a suite of positive attributions (Langlois et al., 2000) and that a positive reputation can benefit one's reproductive fitness (Fehr, 2004; Nowak and Sigmund, 2005), strategic economic behavior in light of a beautiful appearance is to be expected, particularly given the severe penalties incurred when individuals are perceived as having used their looks for nefarious purposes (e.g., in cases of fraud; see Mazzella and Feingold, 1994 for a meta-analytic review; see also Wilson and Eckel, 2006).

If visible cues play an important role in trusting behavior and the exchange of resources, the context in which we interact with others may be important for security-related outcomes. For example, while direct face-to-face combat could be described as the “traditional method” of resource competition, online theft presents an evolutionary-novel challenge that strategists might only just be coming to terms with (see Anderson et al., 2012 for discussion). Given the potential for anonymity in the extortion of resources online, individuals may be better-placed to exploit others with impunity in these contexts. Thus, overconfidence may be expected to “evolve” among hackers, and this may be particularly pronounced among those who are less physically-equipped to inflict *immediate* costs on others during face-to-face competition. Future research could explore the relationship between personality and hacking behavior using a behavioral measure of “persistence” in “code-cracking” tasks.

## Practical applications

Understanding how individual and environmental differences predict security outcomes could generate practical solutions to problems. The extent to which personality and appearance influence social judgments and behavior at key “barriers” to entry may enhance the overall quality of professional recruitment and training. For example, work has already demonstrated that self-rated attention to detail is predictive of security screening performance (Rusconi et al., 2012). In a high-risk, high-reliability industry, stress within the immediate environment may affect the performance of some more than others, even at basic levels of cognition. For example, while experimentally-activating feelings of power has a positive effect on performance in executive-function tasks (Smith et al., 2008) it may also promote abstract thinking at a potential cost of false recognition—broadly speaking, focussing on the bigger picture at the expense of the finer details (Smith and Trope, 2006). The possibility that both transient (following a security breach) and stable (promotion) changes in perceived power within a security role may alter task performance is worthy of further research.

Competition for resources could be investigated at the neural level by exploring the neural basis of individual differences in morality and risk-taking in contexts related to resource acquisition and defence. Testosterone is associated with both financial risk-taking (Apicella et al., 2008; Coates and Herbert, 2008; Stanton et al., 2011) and strict endorsement of utilitarian morals (Carney and Mason, 2010), and increases as feelings of power are primed experimentally (Carney et al., 2010). Individual differences in state and trait levels of testosterone may predict the nature of the “trade-off” between the costs and benefits of monitoring and controlling perceived threats to security. Imaging studies could shed light on this, given that recent work suggests a complementary role for dopamine and noradrenalin in the evaluation of benefit and cost respectively (Bouret et al., 2012).

## Conclusion

Biology provides a unifying framework with which to understand human behavior in light of differences between individuals and their surrounding environment. An understanding of the biological basis of strategic “biases” in social judgments can potentially increase the quality of security decision-making in light of greater awareness of the contexts and environments that might mitigate or exacerbate the risk of lost resources.
